# Assessing Students’ Knowledge on WASH-Related Diseases

**DOI:** 10.3390/ijerph16112052

**Published:** 2019-06-10

**Authors:** Khaldoon A. Mourad, Vincent Habumugisha, Bolaji F. Sule

**Affiliations:** 1 Faculty of Social Sciences, Centre for Middle Eastern Studies, Lund University, 22100 Lund, Sweden; 2 Pan African University Institute of Water and Energy Sciences Including Climate Change, B.P. 119 Pôle Chetouane, Tlemcen 13000, Algeria; vincenthaba@yahoo.fr; 3 Department of Water Resources & Environmental Engineering, University of Ilorin, P.M.B 1515, Ilorin 240003, Nigeria; bfsuleiman@gmail.com

**Keywords:** Rwanda, sanitation, hygiene, education, assessment, waterborne diseases

## Abstract

Water-, sanitation-, and hygiene-related diseases are killing many people each year in developing countries, including Rwanda, and children under the age of five are the most vulnerable. This research assessed human waste disposal practices, knowledge on diseases caused by contact with human faeces, and knowledge on causes and prevention of selected WASH-related diseases. One thousand one hundred and seventy-three students were interviewed out of 2900 students. The results showed, regarding students’ waste disposal practices, that 96.3% use latrines, 20.5% practice open defecation in bushes, and 3.2% defecate in water bodies. Regarding knowledge on diseases caused by contact with human faeces, 56.9% responded that they were aware of cholera, 26.5% of diarrhoea, 2.2% of dysentery, 0.3% of malaria, 0.1% of shigellosis, and 3.8% of typhoid. The majority of the respondents, between 50–99%, could not identify the main causes of the WASH-related diseases. This paper also showed that students lack health knowledge in regard to WASH-related diseases’ causes and prevention. Therefore, the provision of water and sanitation infrastructures should go with the provision of health education on how to avoid these diseases and possible ways to improve the well-being of the students both at home and in their various schools.

## 1. Introduction

Water, sanitation, and hygiene term (WASH) represents a growing movement focusing on improving quality of life by reducing WASH-related diseases [1]. Some of the WASH-related diseases are presented below:

(1) Diarrhoea, which is usually caused by enterotoxigenic *Escherichia coli* [2]. Diarrhoea is responsible for about 21% of all under-five deaths [3]; about three-quarters of a million children die from diarrhoea every year [4]. Inadequate sanitation, hygiene, or access to safe water increase the incidence of diarrhoea diseases and deaths, which mostly occur in developing countries, including most African countries [5,6,7].

(2) Cholera is caused by the bacterium *Vibrio cholera* and is considered a major problem in Africa and Asia. Cholera causes diarrhoea and severe dehydration; sources of infections include standing water, seafood, grains, and unpeeled fruit and vegetables [8]. Overcrowded communities with poor sanitation and unsafe drinking water supplies are more vulnerable to cholera. If untreated, 50% of people with severe cholera will die, but prompt and adequate treatment reduces deaths from severe cholera to less than 1% of cases [5]. Many countries at Sub-Saharan Africa are broadly affected by many cholera cases [9].

(3) Trachoma is an infection caused by *Chlamydia trachomatis* that affects the eyes and may result in blindness. Trachoma has caused the visual impairment of 1.8 million people, with its impacts mostly occurring in developing countries, as poverty, crowded living conditions, and poor sanitation help in spreading the disease [10].

(4) Shigellosis is an intestinal disease caused by a group of bacteria known as *Shigella* and can be stopped by careful handwashing with soap. Infected people may develop diarrhoea, fever, and stomach cramps [11]. Bowen [12] argued that the risk of infection caused by *Shigella* spp. was highest for people traveling to Africa.

(5) Typhoid is caused by *Salmonella typhi* and is usually spread through contaminated water and food. Urbanization and climate change have the potential to increase the global burden of typhoid, which kills around 150,000 people every year [13].

(6) Malaria is on the list of Acquired Immunodeficiency Syndrome AIDS-related opportunistic infections and the highest rates occur in individuals infected with human immunodeficiency virus (HIV) [14]. In pregnant women, the co-infection with malaria and HIV is associated with anaemia, low birth weight, and increased risk of infant mortality to a greater extent than infection with either disease alone [15]. Malaria mortality is usually higher in low-income countries, such as most of the African counties [16]. Malaria is a vector-borne disease that requires better water resources management to reduce its transmission [17].

Improving water, sanitation, and hygiene practices helps in reducing the spread of waterborne diseases [18]. Moreover, ensuring a hygienic environment, clean water, and adequate sanitation can prevent infections associated with HIV/AIDS [19]. The United Nations (UN) identified critical problems associated with WASH-related diseases, especially in developing countries, including those in Africa. Therefore, the United Nations’ Sustainable Development Goal 6: “Ensure access to water and sanitation for all”, aimed at solving these issues by providing safe water and sanitation for all by 2030 because the better management of fresh water and sanitation are the pillars of human health, environmental sustainability, and economic prosperity [20].

Although Rwanda met the Millennium Development Goals (MDGs), the treated wastewaters do not meet the national standards due to poor governmental monitoring, which is considered a big challenge to achieve the Sustainable Development Goals (SDGs) in 2030 [21]. Rwanda faces many barriers to achieving improved sanitation, including the high population density in the cities, the lack of space for latrines in some areas, such as Kigali city, and the costs [22]. The poor hygiene practices and lack of access to improved water and sanitation facilities in Rwanda increases WASH-related mortality and morbidity [23,24,25]. In 2016, the Rwanda Biomedical Centre confirmed the outbreak of non-bloody diarrhoea, typhoid, shigellosis, and cholera cases in Rwanda. According to the World Health Organization and Rwandan Biomedical Centre, flood dispersal of faecal contaminants, water shortages, poor sanitation and hygiene practices, and insufficient knowledge are among the key causes leading to increased risks of outbreaks of waterborne diseases such as cholera [26]. According to the Rwanda Biomedical Centre, from 2010 to 2017, seventeen cholera outbreaks were recorded, with a total of 420 cases and 6 deaths documented.

The main reasons for these outbreaks were drinking from Kivu Lake, poor hygiene, lack of latrines (many households shared one latrine), non-functional water taps, non-chlorinated water, and using surface water as a latrine [26,27].

This paper assesses students’ knowledge on water-, sanitation-, and hygiene-related diseases in randomly selected rural and urban schools in Musanze District in Rwanda, with a focus on human waste disposal practices, diseases caused by contact with human faeces, and the causes and prevention of some WASH-related diseases.

## 2. Methods

### 2.1. Study Area

**Figure 1:**
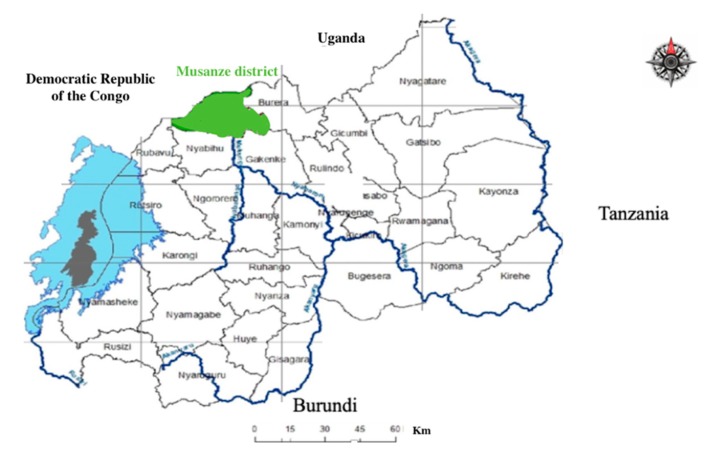
Rwanda and Musanze district location.

Rwanda is surrounded by Uganda, Tanzania, and Burundi. Rwanda is divided into five provinces: City of Kigali, Eastern Province, Northern Province, Western Province, and Southern Province, each of which has three to eight districts [28]. This research was conducted in Musanze District, one of the five districts that make up Northern Province of Rwanda (Figure 1; [29]). The population of Musanze is about 370,000 and a total area of 530.4 km^2^ [30].

### 2.2. Sample Size

Students from six randomly selected schools (three rural and three urban), representing a total student population of 2900, were interviewed. The students’ ages were between 12 and 15 years. Krejcie and Morgan’s [31] table was used to select the sample size from each school, which presents an acceptable margin of error (about 3%).

Krejcie and Morgan used the following formula to determine the sample size [32]:

S = X^2^NP(1 − P)/d^2^(N − 1) + X^2^P(1 − P) (1)

where S is the required sample size; X^2^ is the table value of chi-square for one degree of freedom at the desired confidence level; N is population size; P is the population proportion; d is the degree of accuracy.

Table 1 presents the population size and location for each selected school. All samples were summed up to 1173 students. The data were analysed using Statistical Package of Social Sciences (SPSS) (IBM corporation, Armonk, NY, USA).

### 2.3. Gender and Location

The respondents were 56.6 % females and 43.4% males. In general, there were more females in urban areas (57.7%) compared to rural areas (55.8%). Rural areas appeared to have more students (58.4%) compared to urban areas (41.7%) (Table 2).

## 3. Results and Discussion 

### 3.1. Type of Human Waste Disposal Used by Students

The results showed that, overall, 96.3% of students used latrines as a method of human waste disposal. In rural areas, 97.7% used latrines, while 94.5% used latrines in urban areas. In regard to the students who used bush toilets as a method of human waste disposal, across both rural and urban schools, the results showed that 20.5% used open defecation in bushes, about 31.1% used open defecation in rural areas, while about 5.3% used open defecation in urban areas. On the other hand, the results showed that 3.2% of the students disposed their wastes into water bodies.

### 3.2. Knowledge on Diseases Caused by Contact with Human Faeces

Cholera, diarrhoea, dysentery, shigellosis, and typhoid are diseases connected to contact with human faeces. However, malaria is a water-related disease that is not related to human faeces.

In both rural and urban areas, students mentioned different diseases that they knew were caused by contact with human faeces. Across the schools, 56.9% mentioned cholera, 26.5% mentioned diarrhoea, 2.2 % mentioned dysentery, 0.3% mentioned malaria, 0.1% mentioned shigellosis, 3.8% mentioned typhoid, and 10.1% had no idea. Cholera and diarrhoea were the most mentioned diseases in both rural and urban areas, while shigellosis and malaria were the least mentioned (Table 3). Table 3 also shows that, in general, students in urban areas had more knowledge than those living in rural areas. For example, the differences in the percentages were 5.7 and 7.3 regarding the knowledge of typhoid and diarrhoea, respectively. However, it was the opposite for cholera, with more rural students being knowledgeable of the disease than urban students.

### 3.3. Knowledge on Selected WASH-Related Diseases

#### 3.3.1. Causes

Diarrhoea is a symptom of infection caused by a host of bacterial, viral, and parasitic pathogens living in water contaminated by human faecal and animal faecal matter from municipal sewage, septic tanks, and latrines. Therefore, drinking dirty water that is polluted by human faeces, poor sanitation, and improper hygiene are likely to result in diarrhoea. Amoebas also cause diarrhoea [33]. The bulk of students in both rural and urban areas had no knowledge about the causes of diarrhoea. About 0.2% mentioned that amoebas are the cause of diarrhoea. About 11.3% mentioned drinking dirty water, 15.3% mentioned poor sanitation and hygiene, while 73.1% did not know the causes of diarrhoea. Amoebas and drinking dirty water were mostly stated in both rural and urban schools.

Shigellosis is caused by *Shigella* bacteria. Most of the infections come from the stools or the soiled fingers of an infected person to the mouth of a healthy person. Therefore, contact with human faeces, water pollution by human faeces, and poor disposal of human faeces can result in shigellosis. The bulk of respondents had no idea about the causes of shigellosis. About 0.2% mentioned that it is caused by contact with human faeces, 0.2% responded that it is caused by polluted water, and 1.6% mentioned poor hygiene. However, 98% did not know what causes shigellosis.

Cholera is caused by the bacterium *Vibrio cholera*. People get infected with cholera after eating food or drinking water that has been infected by faeces of a cholera-infected person. Respondents mentioned food and water contaminated by human faeces (10.9%) and *Vibrio cholera* (6.3%). However, 82.8% did not know what causes cholera.

Typhoid is caused by *Salmonella typhi*. People are infected with *Salmonella typhi* via faecal–oral route from infected individuals to healthy individuals. Generally, students had little knowledge about what causes trachoma. Only 0.8% responded that it is caused by washing one’s face with dirty water, while 99.2% did not know the cause. Eating contaminated food or water (4.9%) and *Salmonella typhi* (3.8%) were mentioned to be causes of typhoid. However, about 91.4% did not know the cause of typhoid.

Malaria is caused by plasmodium parasites and is spread when people are bitten by a malaria-infected *Anopheles* mosquito. Female *Anopheles* mosquitoes (58.4%) and stagnant water around home (0.4%) were stated to be causes of malaria. However, about 41.2% did not know the causes of malaria.

#### 3.3.2. Prevention

Having knowledge in the prevention of WASH-related diseases is a key to having better health. About 15.8% of respondents mentioned drinking clean water as a way to prevent cholera, and 37.5% mentioned washing hands with soap and maintaining food hygiene to prevent diarrhoea diseases. About 26.5% stated draining stagnant water, using bed nets, and clearing bushes around the home as a way of preventing malaria. About 0.3% said that they can prevent shigellosis through washing hands with soap and prevention of contact with human faeces, while only 0.1% said that they can avoid swimming in dirty water to prevent trachoma, and about 2.6% said cleaning raw fruits and vegetables and using clean water is the way to prevent typhoid. However, about 17.1% did not have knowledge about how to prevent any of the stated diseases (Table 4).

## 4. Conclusions

The promotion of health and hygiene in schools is essential for all students in order to support the Child-Friendly Schools program [34]. This study focused on assessing student knowledge regarding WASH-related diseases, causes, and prevention. Generally, the bulk of students surveyed used latrines as a method of human waste disposal, however, a small percentage of them used open defection in bushes and water bodies, which can cause the spread of diseases. Many students (10.1%) did not know any disease caused by contact from human faeces, and this can affect their attitudes and practices toward open defecation in bushes and water bodies.

An above average number of respondents did not know the causes of diarrhoea, shigellosis, cholera, trachoma, and typhoid. This is a big problem, because they did not know what their practices and attitudes toward sanitation and hygiene may cause in relation to WASH-related diseases.

We conclude that knowledge on the causes and prevention of WASH-related diseases is very low, and only a very few students knew how to protect themselves and exercise disease prevention. Therefore, the government of Rwanda and partners in water, sanitation, and hygiene should focus not only on providing water and sanitation infrastructures, but also on health education that can raise students’ awareness in regard to the need for individual clean practices and the related risks associated with WASH diseases. The education should be a way to stop open defecation in bushes and water bodies, by teaching students the health risks of diseases caused by contact with human faeces and also teaching students the causes and prevention of WASH-related diseases. This kind of education would result in positive changes in students’ attitudes toward sanitation and hygiene practices. Health education should also include the proper use of sanitation and drinking water infrastructures.

## Figures and Tables

**Table 1:** Schools’ population and sample size.

Name of School	Location of School	Population Size	Sample Size
Esc. Musanze	Urban	208	136
GS ^1^. Muhoza II	Urban	695	248
ESSA Ruhengeri	Urban	140	103
GS. Nyange	Rural	570	226
GS. Kampanga	Rural	912	269
GS. Gakoro	Rural	375	191
Total		2900	1173

^1^ G.S. Group School.

**Table 2:** Gender and locations for students.

			Gender		Total
			Female	Male	
Location of school	Rural	Count	383	303	686
		%	55.8%	44.2%	100%
	Urban	Count	281	206	487
		%	57.7%	42.3%	100%
Total		Count	664	509	1173
		%	56.6%	43.4%	100%

**Table 3:** Knowledge about diseases.

			Cholera	Diarrhoea	Dysentery	Malaria	No Answer	Shigellosis	Typhoid	Total
Location of school	Rural	Count	417	161	13	0	84	1	10	686
		%	60.8%	23.5%	1.9%	0.0%	12.2%	0.1%	1.5%	100%
	Urban	Count	251	150	13	3	35	0	35	487
		%	51.5%	30.8%	2.7%	0.6%	7.2%	0.0%	7.2%	100%
Total		Count	668	311	26	3	119	1	45	1173
		%	56.9%	26.5%	2.2%	0.3%	10.1%	0.1%	3.8%	100%

**Table 4:** Students’ knowledge on WASH-diseases prevention methods.

		Cholera	Diarrhoea	Malaria	No Knowledge	Shigellosis	Trachoma	Typhoid	Total
Rural	Count	86	224	290	74	2	0	10	686
	%	12.5%	32.7%	42.3%	10.8%	0.3%	0%	1.5%	100%
Urban	Count	99	216	21	127	2	1	21	487
	%	20.3%	44.4%	4.3%	26.1%	0.4%	0.2%	4.3%	100%
Total	Count	185	440	311	201	4	1	31	1173
	%	15.8%	37.5%	26.5%	17.1%	0.3%	0.1%	2.6%	100%
